# *In Vivo* Sustained Release of Peptide Vaccine Mediated by Dendritic Mesoporous Silica Nanocarriers

**DOI:** 10.3389/fimmu.2021.684612

**Published:** 2021-06-16

**Authors:** Weiteng An, Sira Defaus, David Andreu, Pilar Rivera-Gil

**Affiliations:** ^1^ Integrative Biomedical Materials and Nanomedicine Laboratory, Department of Experimental and Health Sciences, Universitat Pompeu Fabra, Barcelona, Spain; ^2^ Proteomics and Protein Chemistry Unit, Department of Experimental and Health Sciences, Universitat Pompeu Fabra, Barcelona, Spain

**Keywords:** dendritic mesoporous silica nanoparticles, peptide vaccines, sustained and controlled release, foot-and-mouth disease virus, nanovaccine, immunogenicity, adjuvancy

## Abstract

Mesoporous silica nanoparticles have drawn increasing attention as promising candidates in vaccine delivery. Previous studies evaluating silica-based vaccine delivery systems concentrated largely on macromolecular antigens, such as inactivated whole viruses. In this study, we synthesized dendritic mesoporous silica nanoparticles (DMSNs), and we evaluated their effectiveness as delivery platforms for peptide-based subunit vaccines. We encapsulated and tested *in vivo* an earlier reported foot-and-mouth disease virus (FMDV) peptide vaccine (B_2_T). The B_2_T@DMSNs formulation contained the peptide vaccine and the DMSNs without further need of other compounds neither adjuvants nor emulsions. We measured *in vitro* a sustained release up to 930 h. B_2_T@DMSNs-57 and B_2_T@DMSNs-156 released 23.7% (135 µg) and 22.8% (132 µg) of the total B_2_T. The formation of a corona of serum proteins around the DMSNs increased the B_2_T release up to 61% (348 µg/mg) and 80% (464 µg/mg) for B_2_T@DMSNs-57 and B_2_T@DMSNs-156. *In vitro* results point out to a longer sustained release, assisted by the formation of a protein corona around DMSNs, compared to the reference formulation (i.e., B_2_T emulsified in Montanide). We further confirmed *in vivo* immunogenicity of B_2_T@DMSNs in a particle size-dependent manner. Since B_2_T@DMSNs elicited specific immune responses in mice with high IgG production like the reference B_2_T@Montanide™, self-adjuvant properties of the DMSNs could be ascribed. Our results display DMSNs as efficacious nanocarriers for peptide-based vaccine administration.

## Introduction

Peptide-based vaccines are considered an attractive alternative strategy to overcome many of the limitations of conventional (inactivated, attenuated) whole virus-based vaccines (1–3). They present advantages such as reduced toxicity, good definition of T- and B-cell epitopes for targeted immune responses, cost-effective scale up manufacturing processes, easy handling, storage, and transport (1, 4, 5). These advantages have prompted the progress of many peptide-based vaccines to different preclinical and clinical stages (1, 6, 7). Nevertheless, peptide-based vaccines tend to be poorly immunogenic usually requiring adjuvants, multivalency, and/or delivery systems to become more effective *in vivo*. Adjuvants of different kinds, such as aluminum hydroxide, mineral salts, water-oil emulsions, or liposome-based formulations have been developed to enhance efficacy (7). Although these strategies can boost to a certain extent the low immunogenicity of peptide-based vaccines, only a limited number are approved for human and animal applications due to their not well-established mode of action, as well as to other related toxicity and safety issues (8, 9).

In the last decade, the field of nanovaccines has gained maturity (10–13). Nanoparticles, especially synthetic ones made of polymers, phospholipids, metal, carbon, or silica (14) among other compositions have been extensively studied for vaccine applications ref (1, 9, 15, 16). Within the variety of nanomaterials used for vaccine delivery, mesoporous silica nanoparticles (MSNs), especially dendritic mesoporous silica nanoparticles (DMSNs), are emerging as promising vaccine delivery platforms because of their versatile formulation, boosting abilities, lack of side effects, and depot effect. They have unique central-radial pore structures with large pore sizes (17–19) and are characterized by low cross-linking silica frameworks with fast degradability rate *in vivo* (20). Studies on DMSNS show their enhanced loading capacity, sustained release profile, easy surface functionalization, and potential adjuvant activity (21, 22). Furthermore, DMSNs have shown effective immune potentiation *in vivo*, inducing strong humoral and cellular immune responses against target antigens (23–25). The majority of studies on MSNs-based vaccine delivery systems are focused on carrying large-size immunogens, such as bacterial recombinants, viral capsid proteins and OVA- and BSA-conjugated model vaccines (26–29), whereas few papers explore their use to carry smaller biomolecules, such as peptides in subunit vaccines.

In this study, we extend the use of DMSNs to delivery platforms for peptide-based vaccines and evaluate their *in vivo* effectiveness. We have encapsulated a peptide construct named B_2_T, which confers full protection against foot-and-mouth disease virus (FMDV) in swine (30, 31). Previous publications of the authors have shown that inclusion of a T-cell epitope in the B_2_T construct provides a rather powerful T-cell response (lymphoproliferation, γ-interferon production) (31–33). B_2_T is currently administered emulsified with Montanide™ ISA 50V2 W/O (water in oil) (*i.e.*, B_2_T@Montanide™). This formulation has some drawbacks. For instance, there are several studies reporting unacceptable local reactions toward the Montanide adjuvant (34). Moreover, Montanide requires a dedicated emulsification procedure for each antigen which add complexity to its industrial production (35). To overcome these challenges, we have explored the use of DMSNs loaded with B_2_T as nanovaccine against FMDV. Briefly, we have synthesized DMSNs of different sizes (57 ± 9 nm and 156 ± 10 nm) and have loaded them with B_2_T, naming the resulting nanoformulation B_2_T@DMSNs. Both sizes exhibited high B_2_T loading capacities (570 µg/mg for DMSNs-57 and 580 µg/mg for DMSNs-156) and an *in vitro* sustained B_2_T release profile over 930 h. Furthermore, RAW 264.7 macrophage cells efficiently internalized the fluorescent version of both nanoformulations in a size-dependent manner. Finally, we have confirmed a specific immune response with high IgG production upon vaccination of outbred Swiss mice (Swiss ICR-CD1) with two doses of B_2_T@DMSNs, obtaining similar antibody titers than those elicited by the previous gold standard B_2_T@Montanide™.

## Materials and Methods

For a detailed description of the procedures and more results, we refer the readers to the **Supporting Information File**.

### Synthesis and Characterization of DMSNs-57 and DMSNs-156

The DMSNs with a diameter of 156 nm (designated as DMSNs-156) were synthesized using a modified version of a previously reported method (17). Briefly, 136 mg TEA were added to 50 mL Milli-Q water and stirred at 500 rpm, 80°C for 0.5 h. Then, 760 mg CTAB and 250 mg sodium salicylate (NaSal) was added to the above solution and stirred for another 1 h. Next, 4 ml TEOS was added dropwise to the solution under stirring, which continued overnight. The products were collected by centrifugation at 12,000 rpm for 10 min and washed three times with ethanol. Then, the collected products were extracted three times with 80 ml of methanol solution containing 4.5 ml of HCl (37%) at 65°C for 6 h to remove the template. Finally, the nanoparticles were dried in vacuum at room temperature overnight. DMSNs with a diameter of 57 nm (designated as DMSNs-57) were synthesized following the abovementioned method except for decreasing the amount of structure directing agent NaSal from 250 to 83 mg.

The structure of both DMSNs types was imaged with a transmission electron microscope (TEM, JEOL JEM1010) at an acceleration voltage of 80 kV. TEM specimens were prepared by evaporating one drop of ethanolic nanoparticle solution on Ted Pella Formvar carbon-coated copper grids. The z-potential and hydrodynamic diameter of the samples was determined in a Malvern Zetasizer ZS instrument at 25°C. Samples were dispersed in water and transferred into disposable polystyrene cuvette. The given values are the average of triplicate readings.

See de **Supplementary File** (section *§SI-1.1*) for complementary information.

### B_2_T Synthesis

The dendrimeric B_2_T immunogen was produced as described earlier (31), by conjugation of 2 copies of the B-cell epitope moiety to a maleimide-functionalized T-cell epitope. The conjugation reaction was clean and practically quantitative, and the resulting branched peptide was satisfactorily characterized by HPLC and mass spectrometry. See section *§SI-1.2* for complementary information and section *§SI-2.1* for the synthesis of fluoro-B_2_T@DMSNs.

### B_2_T Loading in DMSNs-57 and DMSNs-156 and Quantification of Peptide Loading

We followed the same methodology to load B_2_T into both DMSNs sizes. The resulting products were named, B_2_T@DMSNs-57 and B_2_T@DMSNs-156. Briefly, 1.5 mg B_2_T and 2.0 mg DMSNs were mixed in 2.0 mL DPBS buffer solution (pH 7.4) and then properly dispersed by sonication for 5 min. The resulting mixture was gently shaken at 200 rpm for 5 h at RT. Afterward, the products were separated by centrifugation at 12,000 rpm for 10 min and washed twice with PBS. B_2_T encapsulation efficiency (EE%) was defined as follows:

$$B_{2}T\text{ encapsulation efficiency }(EE\%)=(weight\;of\;loaded\;B_{2}T/weight\;of\;total\;B_{2}T)\times 100\%$$

where the amount of loaded B_2_T was determined by subtracting the free B_2_T in the supernatant from the total amount, and the amount of free B_2_T in the supernatant was calculated based on the B_2_T calibration curve obtained in DPBS (section *§SI-1.3*, **Figure SI-3**). See section *§SI-1.4* for complementary information on the impact of key parameters (ionic strength, peptide structure, and DMSNs charge) on the loading efficiencies, and section *§SI-2.1* for the loading of fluoro-B_2_T into DMSNs.

### B_2_T Calibration Curve

A B_2_T stock solution (1,000 μg/ml) was prepared by dissolving 2 mg lyophilized B_2_T powder in 2 ml DPBS. From this stock solution serial dilutions in DPBS (31.3, 62.5, 125, 250, and 500 μg/ml) were prepared and measured on a Biochrom™ Ultrospec 2100 Pro UV/Vis spectrophotometer using a quartz cuvette with a 1-cm path length (with DPBS as blank). The calibration curve was constructed by plotting the absorbance at 225 nm against the corresponding B_2_T concentrations. See section *§SI-1.3* for complementary information.

### B_2_T Release Kinetics From the DMSNs

Release experiments were carried out in 1.5 ml Eppendorf tubes containing 1.0 mg DMSNs loaded with B_2_T and 1.0 ml DPBS (pH 7.4). Samples were gently shaken at 37°C and, at predetermined time points, the suspension was centrifuged at 12,000 rpm for 10 min. We took the supernatant and measure the absorbance (225 nm) of B_2_T released. The procedure was repeated for each time point and for both DMSNs. Fresh DPBS (same volume than aliquot of supernatant taken) was added to redisperse the pellet. All release measurements were performed in duplicate.

### Imaging the Cellular Uptake of B_2_T@DMSNs

RAW 264.7 cells in RPMI 1640 medium (containing 2 mM l-glutamine, 10% heat-inactivated FBS, and 1% penicillin and streptomycin) were seeded in Ibidi μ-slide 8 well at a density of 5.0 × 10^4^ cells/well. Cells were incubated at 37°C in an atmosphere of 5% CO_2_ for 24 h. Then, 30 μg/ml of fluoro-B_2_T@DMSNs was added to the cells. Following 0.5, 1, 2, 4, 8, and 16 h incubation, cells were washed three times with PBS, and fresh growth medium containing CellMask deep red plasma membrane was added and incubated for 8 min. After three washes with PBS, fresh PBS was added, and cells were imaged by confocal laser scanning microscopy (CLSM). See section *§SI-2.2* for complementary information.

### Flow Cytometry Analysis of Cellular Uptake

RAW 264.7 cells were seeded in six-well plates in RPMI 1640 medium (containing 2 mM l-glutamine, 10% heat-inactivated FBS, and 1% penicillin and streptomycin) at a density of 1.0 × 10^6^ cells/well. Cells were incubated at 37°C in an atmosphere of 5% CO_2_ for 24 h. Then, 30 μg/ml fluoro-B_2_T@DMSNs was added. Following 0.5, 1, 2, 4, 8, and 16 h incubation, cells were washed three times with PBS, new PBS was added, and cells were carefully detached from the plates with a Falcon cell scraper. The collected cells were transferred to tubes, were placed in ice, and the nuclear dye, DAPI was added to a final concentration of 1.0 µg/ml, and incubated for 2 min. The labelled cells were then measured by flow cytometry (FC) in a BD LSRFortessa X-50 flow cytometer. The mean fluorescence intensity (MFI) and percentage of cells with a positive fluorescent signal compared to the control (untreated cells) were determined on 5,000 gated single-living cells. FACS data were processed by the method described in section *§SI-2.3*.

### Mice Immunization

Experiments were carried out in the animal facility of the CSIC Center for Research and Development (CID-CSIC), in agreement with EU (Directive 2010/63/EU on the protection of animals used for scientific purposes) and domestic (Real Decreto 53/2013) regulations. The protocol to produce antibodies was in accordance with institutional guidelines under a license from the local government (DAAM 7463) and was approved by the Institutional Animal Care and Use Committee at the CID-CSIC.

All formulations were prepared on the day of injection. Mice were randomized into groups and inoculated by two subcutaneous injections over the interscapular area at day 0 and day 21. All mice were euthanized at day 40 by carbon dioxide inhalation. Animals were monitored three times per week for health during the study.

To assess immunogenicity of B_2_T@DMSNs in mice, two trials were performed (section *§SI-3)*. In the first one, mice were divided into three groups as shown in **Table SI-1**. The first group was the positive control group (4 mice) which was immunized with 200 µl of Montanide ISA 50V2 emulsion containing 100 µg B_2_T (B_2_T@Montanide™), following earlier studies (30); the second (six mice) and third (four mice) groups were the sample groups. The second group was treated with 100 µg B_2_T loaded in DMSNs-156 (B_2_T@DMSNs-156) in 200 µl DPBS, and the third group was treated with the same amount of DMSNs alone (163 µg DMSNs-156) in 200 µl DPBS. All groups were boosted at day 21. Blood samples were collected before vaccination (day 0) and at days 14, 20 (pre-boost), and 40 (euthanize, sample obtained by cardiac puncture). In the second trial, aimed at assessing the impact of DMSNs size on mice immunization, mice were divided into three groups as shown in **Table SI-2**. The first group was again the positive control group (B_2_T@Montanide™; three mice). The second (five mice) group was treated with the formulation B_2_T@DMSNs-57 and the third (five mice) group with B_2_T@DMSNs-156. All mice were treated with the same dose of peptide vaccine (100 µg B_2_T). Blood sample collection was extended until day 80, to study the long-term immune effect of B_2_T@DMSNs.

### Detection of Specific Anti-B_2_T Antibodies by ELISA

Specific antibodies were detected by enzyme-linked immunosorbent assay (ELISA). 96-well Costar^®^ plates were coated with 50 µl B_2_T (15.4 µg/ml) in bicarbonate/carbonate coating buffer (0.05 M, pH 9.6) and incubated at 4°C overnight. After washing three times with DPBS, 50 µl of diluted serums (two-fold dilution series of each collected serum sample were prepared, starting at 1/150, and each dilution sample in duplicate) were incubated for 1 h at 37°C, followed by four DPBS washes. Pre-immune sera from mice were used as negative controls. Next, 50 µl of a 1:4,000 dilution of HRP-labeled rabbit anti-mouse IgG were added and incubated for 1 h at 37°C followed by five washings with DPBS. Then, 100 µl of TMB substrate solution was added for 20 min at RT in the dark. Finally, the reaction was stopped by adding 100 µl of 1 M H_2_SO_4_. The optical density (OD) of the samples was measured in an ELISA reader (BioRad, CA, USA) at 450 nm. Titers in a log10 scale were expressed as the reciprocal of the last dilution giving the absorbance recorded in the control wells (serum at day 0) plus 2 SD. See section *§SI-3.2* for complementary information on the individual response of each mice to the treatment.

### Statistical Analysis

Differences among B_2_T@DMSNs-immunized groups in B_2_T-antibody titers were analyzed by one-way ANOVA, followed by Tukey’s post-hoc comparisons tests. Values are cited in the text as means ± SD. All p values are two-sided, and *p* values < 0.05 were considered significant. Statistical analyses were conducted using GraphPad Prism Software 5.0 (San Diego, CA, USA).

## Results

### DMSNs Synthesis and Physicochemical Characterization

**Figure 1:**
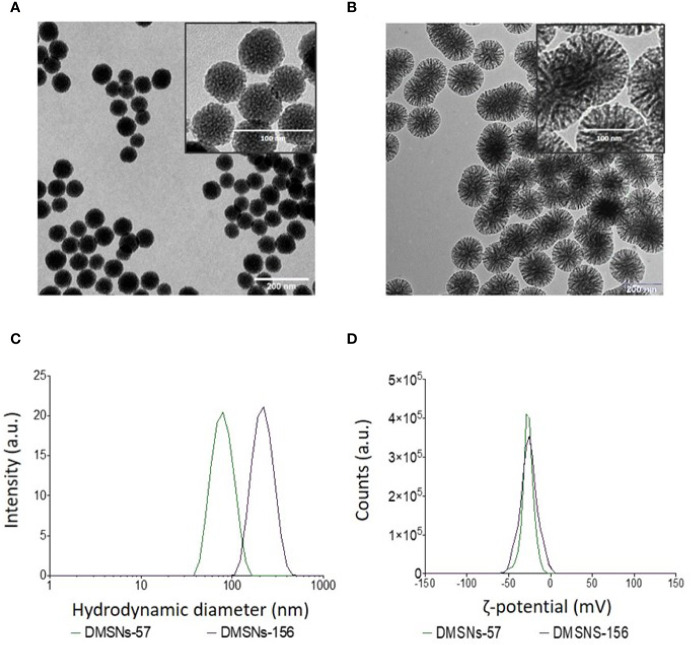
TEM and DLS analysis of DMSNs-57 and DMSNs-156. TEM image of 57 nm DMSNs **(A)** and of 156 nm DMSNs **(B)**. Scale bar, 200 nm. The insets show the DMSNs with higher magnification revealing the dendritic structure. Scale bar, 100 nm. **(C)** DMSNs-57 and DMSNs-156 hydrodynamic size (75 ± 9 nm and 156 ± 10 nm) and **(D)** ζ-potential values (-30.2 and -37.1 mV).

TEM measurements showed that both types of DMSNs have an inorganic core diameter of 57 ± 9 nm (DMSNs-57) and 156 ± 10 nm (DMSNs-156) (**Figure 1A, B**; *§SI-1.1*, **Figures SI-1A–D**). DLS measurements indicated that the averaged hydrodynamic diameter of DMSNs-57 was 75 nm with a polydispersity index (PDI) of 0.060 and the averaged hydrodynamic diameter of DMSNs-156 was 227 nm with a PDI of 0.061 (**Figure 1C**; §SI-1.1, **Figure SI-1.E**). The low PDIs for both nanoparticles demonstrate excellent monodispersity and uniformity which are consistent with TEM images. As expected, the DLS measurement showed higher size values for the DMSNs than those measured by TEM. This is due to the DMSNs’ surface hydration in aqueous solution (36). The z-potential values of DMSNs-57 and DMSNs-156 were -30.2 mV and -37.1 mV, respectively (**Figure 1C**). These results indicate colloidal stability and homogenous size distribution.

### Loading B_2_T Vaccine Into Differently Sized DMSNs (B_2_T@DMSNs) and *In Vitro* Characterization of B_2_T Release Kinetics

After synthesizing DMSNs-57 and DMSNs-156, we performed their loading with the B_2_T peptide vaccine (see section *§SI-1.2*, **Figure SI-2** for B_2_T structure). The B_2_T amount loaded into both types of DMSNs was quantified based on its absorbance at 225 nm (section *§SI-1.3*, **Figure SI-3A**) and using a calibration curve (section *§SI-1.3*, **Figure SI-3B**). We quantified 1.14 mg and 1.16 mg of B_2_T loaded in 2.0 mg of DMSNs-57 and DMSNs-156, respectively. The loading capacities were 570 µg/mg DMSNs for DMSNs-57 and 580 µg/mg DMSNs for DMSNs-156, and the encapsulation efficiencies (EE%) reached 76% and 77%, respectively. Regardless the differences in DMSNs sizes, we measured similar loading efficiencies. We attribute this to their close z-potential values and to the equivalent hydrogen bonds and polar interactions with the peptide (37). The high B_2_T loading capacities obtained are probably related to the strong electrostatic interaction between the anionic DMSNs and the positively charged B_2_T (pI 10.88) in DPBS (pH 7.4) and to the DMSNs central-radial pore structures with large surface areas (17, 18).

We performed the loading under different conditions (section *§SI-1.4*) (38, 39) to evaluate the impact of ionic strength (**Figure SI-4**), peptide (cargo) structure (dendrimer *vs.* linear) (**Figure SI-5**) and DMSNs charge (**Figure SI-6**) on the loading efficiency of the DMSNs. We used, for comparison, 168 nm solid silica nanoparticles (SNSs-168) (**Figures SI-4** and **SI-5**). Results on section *§SI-1.4* (**Figures SI-4, SI-5, SI-6**) displayed that the higher the ionic strength, the more B_2_T was loaded into all silica nanoparticles. Being DMSNs more efficient than SNSs. The trend was maintained for the dendrimer B_2_T and a linear control peptide (O PanAsia B epitope B) regardless of DMSNs charge. Up to 5× ionic strength, DMSNs-156 were more efficiently loading the peptide. Note that within this work the ionic strength was set at 1×. Furthermore, we observed that our synthesized, negatively charged DMSNs were significantly more effective in loading the B_2_T peptide than their positively charged counterparts (**Figure SI-6**).

**Figure 2:**
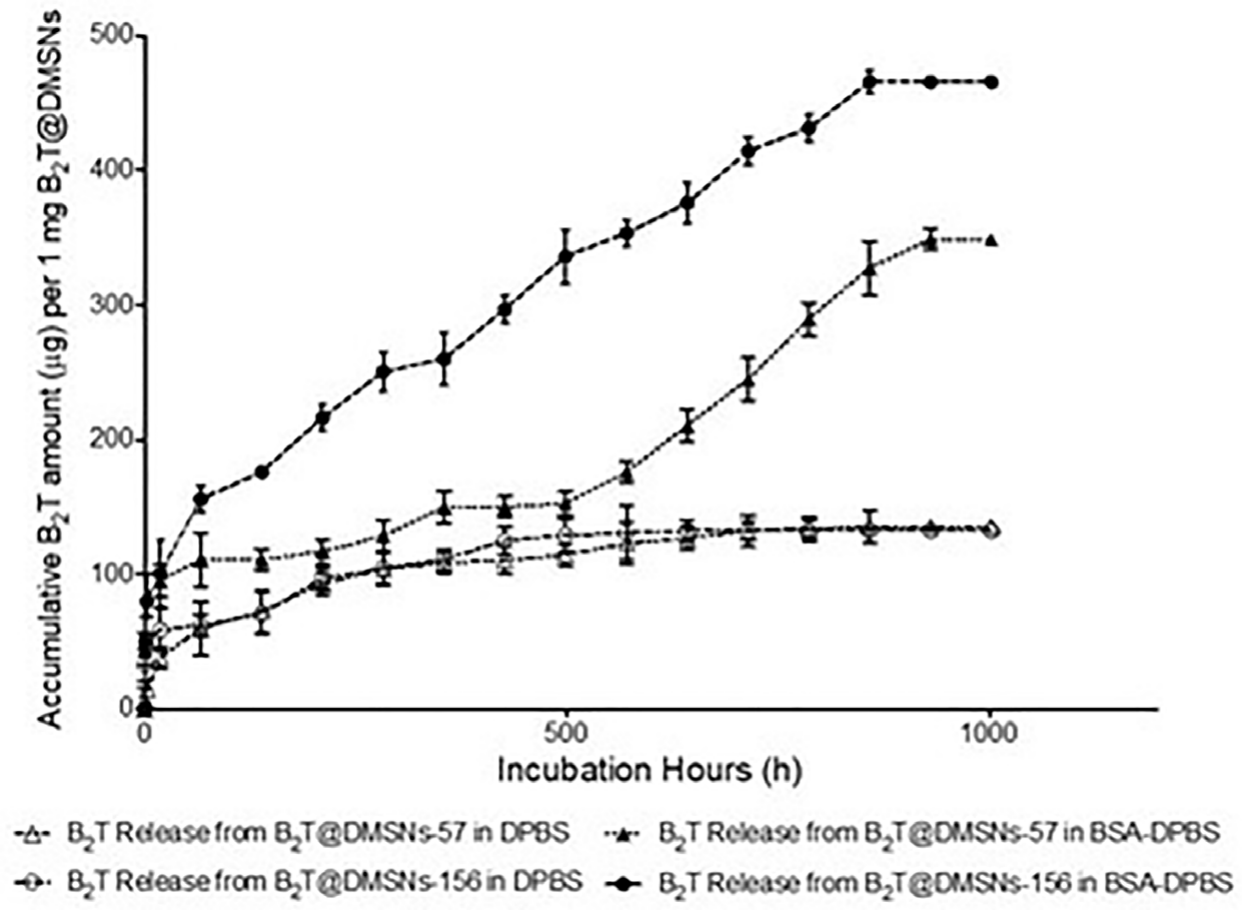
B_2_T release profiles from B_2_T@DMSNs-57 and from B_2_T@DMSNs-156 dispersed in DPBS or BSA-DPBS. After each time point, the supernatants were collected for UV-vis analysis and the pellets were redispersed in the same volume of medium. The procedure was repeated during 1,000 h.

Next, we investigated the B_2_T release kinetics from the DMSNs. To this end B_2_T@DMSNs were dispersed in a saline buffer (1× DPBS). At given time points, we collected the supernatants after centrifugation, we measured their absorbance at 225 nm and with help of the calibration curve (**Figure SI-3.B**), we quantified the amount of B_2_T released from the DMSNs. **Figure 2** shows a sustained release of B_2_T up to 1000 h (41 days). After 700 h, the release curve reached a plateau. Both B_2_T@DMSNs-57 and B_2_T@DMSNs-156 showed similar release kinetics. The B_2_T amount released in B_2_T@DMSNs-57 and B_2_T@DMSNs-156 corresponds to 23.7% (135 µg) and 22.8% (132 µg) of the total amount loaded.

Albumin is one of the most frequent proteins in physiological fluids and a major component of the protein corona of biomedical nanomaterials dispersed in such fluids (40–44). It is also known that the protein corona formed on nanoparticles is a dynamic system. Following typical nanoparticle behavior, we expected a protein corona around our DMSNs upon their *in vivo* administration. We therefore wanted to elucidate the impact of the protein corona on the B_2_T release kinetics (**Figure 2**). To this end, we dispersed the B_2_T@DMSNs in medium containing albumin (BSA 250 µg/ml in DPBS), allowed the DMSNs to build their protein corona and measured the B_2_T release (section *§SI-1.5*) following the procedure described before. We took advantage of the distinct absorption peaks for B_2_T at 225 nm (section *§SI-1.3*) and for albumin at 280 nm (section *§SI-1.5*, **Figure SI-7**) to build calibration curves. In this case we could also track changes on the protein corona formed around the B_2_T@DMSNs. Our methodology enabled the concomitant quantification of the release of both components, B_2_T and albumin, from the DMSNs to the medium. We validated this technology with HPLC (section *§SI-1.5*, **Figure SI-8**). Then we quantified the B_2_T release from the protein coated DMSNs (**Figure 2**) and correlated the results with the amount of albumin released from the protein corona (section *§SI-1.5*, **Figure SI-9**).

After the formation of the protein corona, B_2_T release increased 158% on B_2_T@DMSNs-57 and 252% on B_2_T@DMSNs-156. This corresponds to 61% (348 µg/mg) and 80% (464 µg/mg) of the total B_2_T loaded within B_2_T@DMSNs-57 and B_2_T@DMSNs-156, respectively. It seems evident, that the presence of BSA significantly enhances B_2_T release. We ascribed this effect to a competitive interaction towards the DMSNs in favor of BSA resulting in B_2_T displacement and release (45, 46). To prove this, we monitored the changes of BSA concentration in the dispersed medium in the presence of the DMSNs (**Figure SI-9**). As seen in **Figure SI-9**, during the first 66.5 hours, both B_2_T@DMSNs-57 and B_2_T@DMSNs-156 kept absorbing BSA from the medium, probably due to forming BSA protein corona on DMSNs, which resulted in lower BSA concentrations (< 250 µg/mL) in the supernatants. Afterwards, the BSA level in both formations kept fluctuating around 250 µg/ml (initial concentration added) which points out to an absence of protein corona around the DMSNs. Although longer experiments would be required to draw a conclusion, these results may indicate a long-term sustained release promoted by the DMSNs. At any rate, they confirm vaccine release from the DMSNs in physiological complex media as the one in the cell.

### Internalization of B_2_T@DMSNs by Macrophages

**Figure 3:**
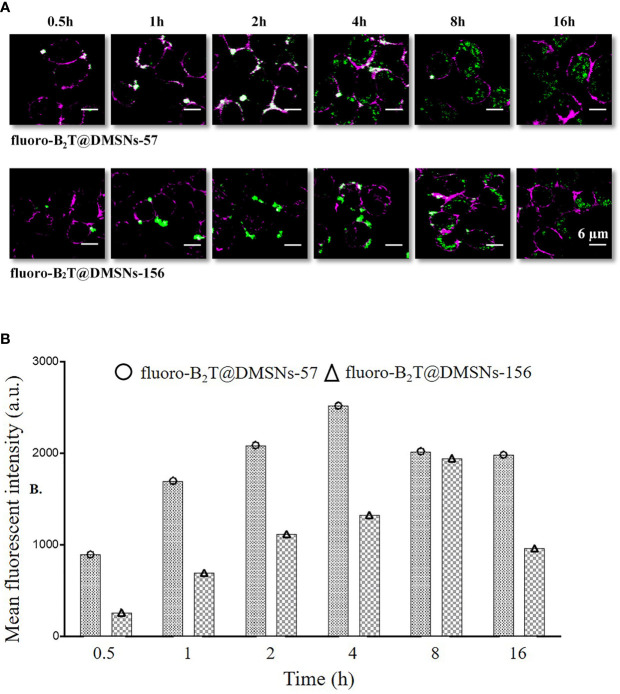
RAW 264.7 macrophage cellular interactions of fluoro-B_2_T@DMSNs-57 and fluoro-B_2_T@DMSNs-156. **(A)** CLSM images showing a time- and DMSNs size-dependent internalization. (Note: Green correspond to BodiFluor-488 conjugated to the B_2_T loaded within the DMSNs whereas the magenta color corresponds to the dye, cell mask deep red used to stain the plasma membrane of the cells). (*cf. §*SI-11, **Figures SI-11, SI-12**, and **SI-16**) **(B)** Flow cytometry analysis of cellular interactions. The columns represent the mean fluorescence intensity of fluoro-B_2_T@DMSNs-57 and fluoro-B_2_T@DMSNs-156. (*cf.* §SI-2.3, **Figures SI**–**13**).

Cellular uptake of antigens by innate immune cells provides antigen-processing and subsequent costimulatory signals that are crucial to trigger acquired immune responses, especially for low immunogenic peptide antigens. Macrophage-like RAW 264.7 cells (47) are often used to study cellular responses to microbes and their products (48). We selected this cell model to assess *in vitro* cellular internalization of our nanoformulations, using 1 mg DMSNs-57 and 1 mg DMSNs-156 loaded with 200 µg B_2_T labeled with a dye (*i.e.*, fluoro-B_2_T) (see section *§*SI-2 and **Figure SI-10**). Similar to other nanoparticles (49), cellular uptake of fluoro-B_2_T@DMSNs occurred in a size-dependent manner (**Figure 3**). The maximum uptake level was observed after 4 h for the fluoro-B_2_T@DMSNs-57 (57 nm size) and after 8 h for the fluoro-B_2_T@DMSNs-156 (156 nm size) (**Figures 3A, B**, and *§*SI-2 and **Figures SI-11, SI-12** and **SI-13**). During the first 4 h, the amount of B_2_T@DMSNs-57 interacting with the cells was approximately two times the amount of B_2_T@DMSNs-156 (**Figure 3B**). We can conclude that at least after an acute exposure, the smaller DMSNs-57 are faster internalized by RAW 264.7 cells than larger DMSNs-156. It is noteworthy that after the cellular uptake reached the maximum value, longer incubation times resulted in reduced uptake values. We suppose that it is due to the fast cell growth and division of RAW 264.7 cells (50) which resulted in the “dilution effect” of fluorescence intensity per cell.

### Sustained Mice Immunogenicity Provided by B_2_T@DMSNs

**Figure 4:**
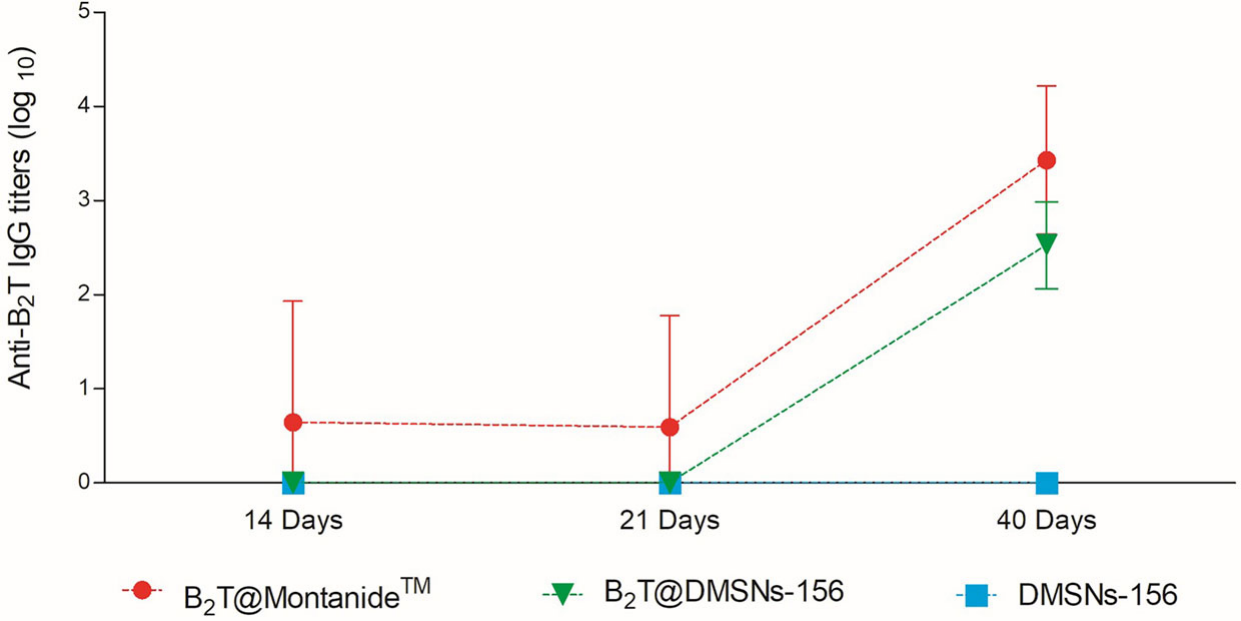
*In vivo* functional validation. ELISA-determined anti-B_2_T peptide responses of mice vaccinated with B_2_T@Montanide™ (red circle, n=4), B_2_T@DMSNs-156nm (green down triangle, n=6), DMSNs-156nm (blue squares, n=4) from sera collected at days 14, 21 (pre-boost) and 40 (post-boost) post-immunization. Each point depicts mean antibody titers (calculated as described in Materials and methods) ± SD for each group. No individual spontaneous reactivity was observed in the titers determined at day 0. (*cf.* §SI-3; **Table SI-1**, and **Figures SI-14, SI-15**, and **SI-16**).

We next validated B_2_T@DMSNs performance by testing *in vivo* their immunogenicity. To this end, we performed two sets of vaccination trials in mice (see section *§*SI-3 for a detailed description). In both trials, we injected subcutaneously samples containing the same amount of B_2_T antigen (100 µg) at day 0 and boosted with the same dose at day 21. We performed an ELISA to detect specific anti-B_2_T antibodies in sera collected following the schedule shown in **Tables SI-1** and **SI-2** (section *§*SI-2). In the first trial (**Table SI-1** and **Figure 4**), mice were vaccinated with B_2_T@Montanide™ (positive control), B_2_T@DMSNs-156, and bare DMSNs-156 (negative control). Results in **Figure 4** show that B_2_T@DMSNs treatment elicits a consistent response with all treated mice, presenting an increase in anti-B_2_T IgG production values after the boost (day 40). Although the anti-B_2_T IgG level from B_2_T@DMSNs-156 is slightly lower than B_2_T@Montanide, these results confirm that B_2_T@DMSNs-156 successfully stimulates anti-B_2_T–specific immune response in mice. On the contrary, as expected, no enhancement of the immune response was found in mice treated with bare DMSNs-156.

**Figure 5:**
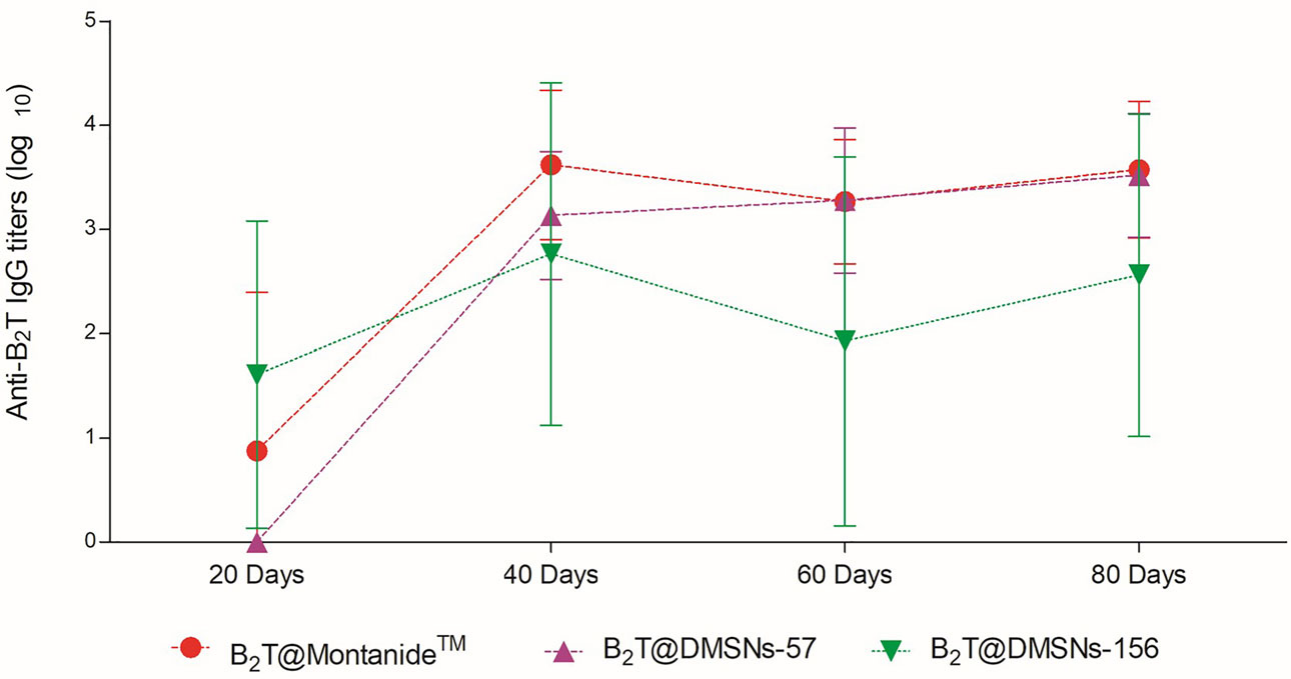
Sustained *in vivo* immune response performed by the DMSNs. ELISA-determined anti-B_2_T peptide responses obtained in vaccination trial II (**Table SI-2**) of mice vaccinated with B_2_T@Montanide™ (red circle, n=3), B_2_T@DMSNs-57nm (purple up triangle, n=5) and B_2_T@DMSNs-156nm (green down triangle, n=5) from sera collected on the indicated days post-immunization (20, 40, 60, and 80 pi). Each point depicts mean antibody titers (calculated as described in Materials and methods) ± SD for each group. No individual spontaneous reactivity was observed in the titers determined at day 0. (*cf.* §SI-3; **Table SI-2, and Figures SI-14, SI-15, and SI-16**).

Once we confirmed the immunogenic effect of B_2_T@DMSNs and considering their long-time sustained release profile obtained *in vitro* (**Figure 2**), we performed a second trial (section *§*SI-2, **Table SI-2**). In this case, mice vaccinated with either B_2_T@DMSNs-57 or B_2_T@DMSNs-156 particle sizes were subjected to a longitudinal analysis of serum-IgG responses up to 80 days. As shown in **Figure 5**, anti-B_2_T IgG titers were clearly boosted up among all tested formulations at day 40, although this time we also detected serum-IgG responses in some mice immunized with B_2_T@DMSNs-156 already at day 20 before the boost. We do not have a clear explanation for these different results between trials, so we attribute it to the intrinsic variability of *in vivo* studies (section *§*SI-3, **Figures SI-14, SI-15**, and **SI-16**). B_2_T@DMSNs-57 and B_2_T@DMSNs-156 showed slightly lower post-boosting titers than the positive control, B_2_T@Montanide™. However, in the case of the B_2_T@DMSNs-57 mice group, their serum titers increased over time until reaching comparable IgG levels to the positive control group at days 60 and 80 with high consistency among individuals. These results with the DMSNs-57 formulation are in consonance with published works reporting nanoparticle traffic to the draining lymph node in a size-dependent manner, with small 20~50 nm nanoparticles being more efficiently drained than bigger ones (9, 23, 25). We can confirm the efficiency of DMSNs to induce sustained Ab responses in a size dependent manner comparable to the emulsified version B_2_T@Montanide™, pointing to demonstrable adjuvant properties of DMSNs. Finally, it is worth noting that, as not all B_2_T is released from the DMSNs at day 80, one could possibly expect a sustained immunogenic effect beyond that time point.

## Conclusions

Biopharmaceutical companies are now actively focused on the development of sustained release drug delivery systems, in view of their inherent benefits. Sustained release formulations designed to maintain the required therapeutic concentrations over an extended period of time present several advantages over conventional dosage forms, including less frequent drug dose, reduced concentration fluctuations, minimal side effects, reduced healthcare costs, improved efficiency and/or immune responses (51, 52). In this context, DMSNs are gaining increasing interest as effective delivery system because they are tunable, exhibit high loading capacity for therapeutic agents, and their release can be controlled. In this work, we evaluate the applicability of these nanocarriers in vaccination and long-term protection using a peptide-based vaccine with previously reported protective immunity against FMDV. Our results demonstrate that DMSNs are colloidally stable and monodisperse, with high loading capacities for a bioactive peptide such as B2T, besides being reported as non-toxic (53–56). The B_2_T@DMSNs resulting formulations present long-term sustained *in vitro* release properties, enhanced in the presence of BSA. Tracking a fluoro-labeled version of B_2_T within DMSNs formulations we could observed acute differences (within 16 h) in the internalization of the B_2_T@DMSNs by macrophage cells in a size dependent manner. Finally, the effectivity of B_2_T@DMSNs as nanovaccine was validated *in vivo* by comparing the inmunogenic response to that of the positive control B_2_T@Montanide™. Mice vaccination trials showed that both DMSNs formulations increased specific B_2_T antibody titers in a similar manner. However, results revealed a trend toward higher antibody titers in the animal group immunized with DMSNs of smaller particle size (57 nm) in agreement with previous literature (57, 58). Taken together, these results indicate that DMSNs is an excellent carrier for peptide vaccine which favors the internalization of the antigen by immune cell. Besides, they also delay or slown down their *in vivo* release, finally leading to a long-lasting sustained immune response activation. Therefore, DMSNs may be a suitable vaccine delivery system alternative to conventional adjuvanted vaccines not only for whole viruses or protein antigens but also for synthetic peptide-based subunit candidates.

## Data Availability Statement

The original contributions presented in the study are included in the article/**Supplementary Material**. Further inquiries can be directed to the corresponding author.

## Ethics Statement

The animal study was reviewed and approved by DAAM 7463.

## Author Contributions

WA contributed to the design of the synthesis study, to data analysis, and wrote the first draft. SD contributed to the design of the *in vivo* study, and to its data analysis. DA contributed to the design of the *in vivo* study. PR contributed to the conception and design of the study, to data analysis and wrote the manuscript. All authors contributed to the article and approved the submitted version.

## Funding

This research was funded by Spanish Ministry of Science, Innovation and Universities (grants AGL2014-48923-C2 and AGL2017-84097-C2-2-R to DA) as well as by Generalitat de Catalunya (grant 2009SGR492 to DA).

## Conflict of Interest

The authors declare that the research was conducted in the absence of any commercial or financial relationships that could be construed as a potential conflict of interest.
