# Supplementary material for: In Vivo Sustained Release of Peptide Vaccine Mediated by Dendritic Mesoporous Silica Nanocarriers
Source: Front Immunol. 2021 Jun 16;12:684612. doi: 10.3389/fimmu.2021.684612 (PMC8244784; doi:10.3389/fimmu.2021.684612)
Supplement: Supplementary file 1 [file DataSheet_1.docx]

**SUPPLEMENTARY INFORMATION**

***In vivo* sustained release of peptide vaccine mediated by dendritic mesoporous silica nanocarriers**

Weiteng An^1,2^, Sira Defaus^2,*^, David Andreu^2^, Pilar Rivera Gil^1,*^

^1^ Integrative Biomedical Materials and Nanomedicine Lab, Department of Experimental and Health Sciences, Universitat Pompeu Fabra, Barcelona 08003, Spain

^2^ Proteomics and protein chemistry unit, Department of Experimental and Health Sciences, Universitat Pompeu Fabra, Barcelona 08003, Spain

Contents

[Section SI-1. Synthesis and physicochemical characterization of B_2_T@DMSNs. 2](#_Toc67396271)

[1.1 Characterizations of DMSNs-57 and DMSNs-156 by TEM and DLS. 2](#_Toc67396272)

[1.2 B_2_T peptide. 3](#_Toc67396273)

[1.3 B_2_T calibration curve in DPBS. 3](#_Toc67396274)

[1.4 Effect of DMSNs charge, peptide structure and medium ionic strength on the loading efficiency. 4](#_Toc67396275)

[1.5 Tracking changes on theprotein corona formed around B_2_T@DMSNs during peptide release. 6](#_Toc67396276)

[Section SI-2. Internalization of B_2_T@DMSNs by mice macrophages. 9](#_Toc67396277)

[2.1 Synthesis of fluoro-B_2_T@DMSNs. 9](#_Toc67396278)

[2.2 Confocal laser scanning microscopy (CLSM) of cellular internalization. 10](#_Toc67396279)

[2.3 Flow cytometry analysis of DMSNs internalization by Raw 264.7 macrophage cells. 12](#_Toc67396280)

[Section SI-3. Mice immunogenicity provided by B_2_T@DMSNs. 14](#_Toc67396281)

[3.1 Vaccination trials (I and II). 14](#_Toc67396282)

[3.2 Individual mice response to vaccination in trial II. 16](#_Toc67396283)

[References. 17](#_Toc67396284)

# Section SI-1. Synthesis and physicochemical characterization of B_2_T@DMSNs.

## Characterizations of DMSNs-57 and DMSNs-156 by TEM and DLS.

**Figure SI-1.** (A, B) TEM images of DMSNs-57. (C, D) TEM images of DMSN-156. (E) DLS analysis results of DMSNs-57 and DMSNs-156.

## B_2_T peptide.

A dendrimer peptide construct termed B_2_T, consisting of two copies of a B-cell epitope [VP1(140-158)] linked through maleimide groups to a T-cell epitope [3A(21-35)] from foot-and-mouth disease virus (FMDV) serotype O-UKG 11/01 was synthesized following conjugation in solution of the linear peptide modules previously assembled by solid phase synthesis and purified to homogeneity by HPLC. A Lys-Lys dipeptide motif was included to define a cleavage site for cathepsin D, a protease putatively involved in *in vivo* antigen processing for presentation to the MHC class II molecules[1]. The B_2_T construct was obtained in a clean, efficient thiol–maleimide ligation at pH 6.0, purified by HPLC and characterized by mass spectrometry[2][3].

**Figure SI-2.** Amino acid sequence and structure of B_2_T.

## B_2_T calibration curve in DPBS.

Standard curves for peptide/protein are normally plotted based on the absorbance of the aromatic amino acids (Trp or Tyr) at 280 nm wavelength. B_2_T peptide lacks of these amino acids (Fig. SI-2) and therefore has no absorbance at 280 nm (figure SI-3.A). Instead, the B_2_T peptide bond absorbs at 225 nm (Fig. SI-3.A) [38]. We therefore plot the B_2_T absorbance at 225 nm *vs.* B_2_T concentration and obtained B_2_T calibration curve (Fig. SI-3.B). The regression equation obtained was *y = 3.5339x + 0.0337*, where *y* is absorbance value at 225 nm and *x* is B_2_T concentration (mg/mL) in 1X DPBS. The coefficient of determination was *r^2^=0.9975*.

**Figure SI-3.** (A) Absorption spectrum of B_2_T in 1X DPBS. (B) B_2_T standard curve based on its absorbance at 225 nm.

## Effect of DMSNs charge, peptide structure and medium ionic strength on the loading efficiency.

*Ionic strength.*

At first, we evaluated the effect of the ionic strength on the capacity of DMSNs-57 and DMSNs-156 to load B_2_T. For comparison, we used non-porous silica nanospheres with a size of 168 nm (SNSs-168) and a similar ζ-potential (-33.9 mV) value to the DMSNs. The loading experiments were carried out with 0.35 mg silica NPs and 0.35 mg lyophilized B_2_T in 0.35 ml DPBS with different ionic strengths. Figure SI-4 shows how the higher the salt content (ionic strength), the higher the loading capacities of all three kinds of silica NPs. In MilliQ water (no salt contents, 0X), all silica NPs displayed negligible amount of B_2_T loading, however in 0.5X DPBS, the loaded B_2_T significantly increased among all NPs. At low ionic strength, (0.5X and 1X), the loading seems to correlate with the NPs pore volumes. SNSs-168, exhibiting lower pore volume, showed lowest amounts of loaded B_2_T compared to the DMSNs. In contrast, at high ionic strength (5X and 10X), all three silica NPs showed similar B_2_T loading capacities. This result highlights the importance of ionic strength on B_2_T loading efficiencies.

**Figure SI-4.** Effect of ionic strength *i.e.,* 0X DPBS (MilliQ water), 0.5X DPBS, 1X DPBS, 5X DPBS, and 10X DPBS on the B_2_T loading capacity by different silica NPs *i.e.,* DMSNs-57, DMSNs-156 and SNSs-168. Note that our working concentration within this work is 1X DPBS.

*Peptide structure.*

We further wonder whether the DMSNs loading capacities could be affected by the molecular structure of the peptide construct. In this regard, we performed the loading of the DMSNs with the linear B-epitope dendrimer precursor (designated as OpanAsia B, Fig. SI-2) in the three types of silica NPs, following the same procedure. Figure SI-5 shows that all silica NPs with an ionic strength ranging from 0 to 5X DPBS loaded significantly higher amounts of B_2_T than OpanAsia B. At higher salt content (10X DPBS) both DMSNs were loaded with similar amounts of both peptides whereas SNSs-168 kept the trend.

*DMSNs charge.*

**
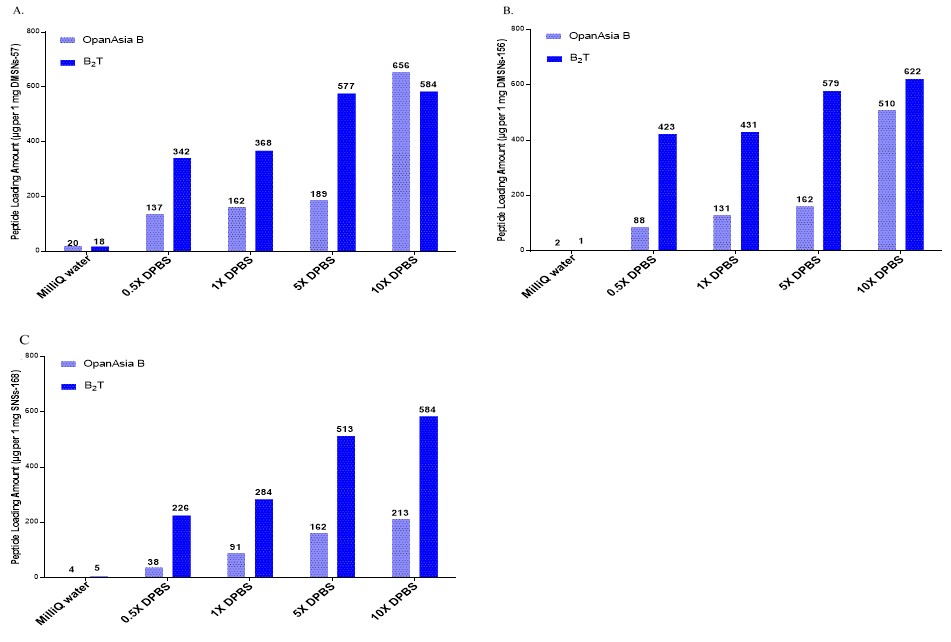
**

**Figure SI-5.** Loading of B_2_T dendrimer and the former linear epitope OpanAsia B by DMSNs-57 (A), DMSNs-156 (B), and by SNSs-168 (C) in the absence (MilliQ water) and presence of salt (0.5X – 10X DPBS).

Our as synthesized DMSNs have a negative ζ-potential value (-37.1 mV). To establish the impact of charge on peptide loading, we synthesized amino-modified DMSNs-156 (designated as DMSNs-NH_2_-156). We followed the method described by Tu *et al*.[4]. The ζ-potential was reversed from -37 mV to +34 mV. We could not observe changes in their dendritic structure and size under TEM and DLS (data not show). Figure SI-6 shows that positively charged DMSNs-NH_2_-156 loaded less amounts of the linear peptide than their negative counterparts, DMSNs-156, under all ionic strengths. Still the effect was more significant at high ionic strength (10X).


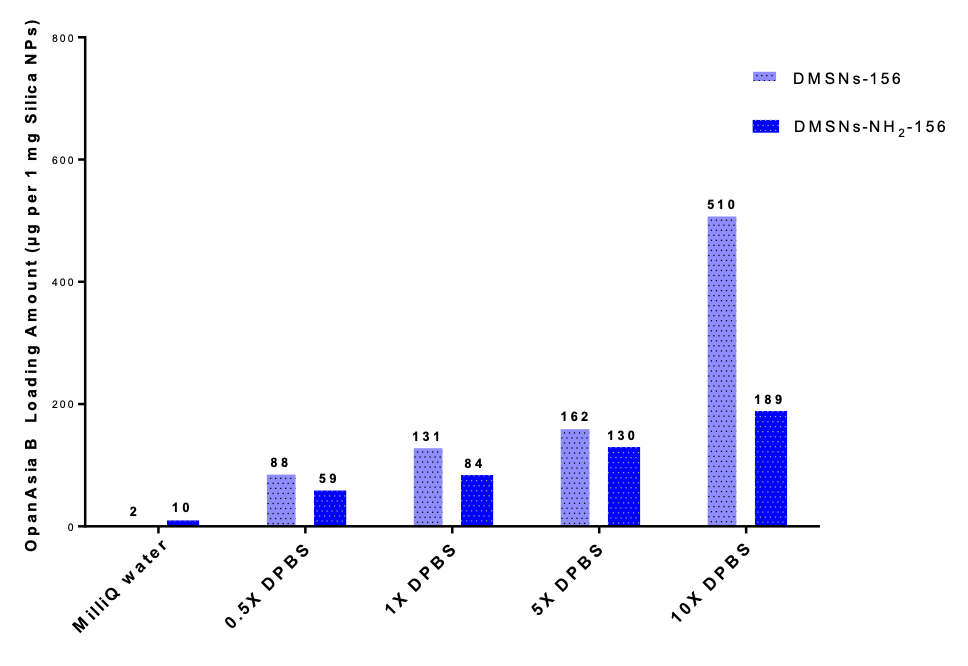


**Figure SI-6.** Comparison of OpanAsia B loading under different ionic strengths (from 0 to 10X DPBS) by negatively (DMSNs-156) and positively charge (DMSNs-NH_2_-156) DMSNs.

We conclude that the efficiency of peptide loading by silica nanoparticles depends on the ionic strength. High salt content significantly enhanced peptide loading capacity irrespective of nanoparticles porosity. Additionally, negatively charged DMSNs carry more peptide than their positively charged counterparts. Under regular working conditions (1X salt content), the dendrimer peptide was better loaded to the DMSNs than one of its linear peptides building blocks.

## Tracking changes on theprotein corona formed around B_2_T@DMSNs during peptide release.

Most NPs in physiological media form a corona of proteins around them. Therefore, for controlling the release of cargo from nanoparticles, we must study the influence of this corona on the B_2_T release kinetics.

*UV-Vis calibration curve for albumin*.

Apart from tracking B_2_T release from the protein coated DMSNs, we also quantified the amount of albumin (major component of the protein corona) detached from the protein corona to the medium. To achieve this, we first prepared a calibration curve for albumin. A BSA stock solution (500 μg/mL) was prepared by dissolving 1 mg BSA powder in 2 mL DPBS. From this stock solution, dilution series in DPBS (50, 100, 150, 250 and 350 μg/mL) were prepared and measured on a Biochrom™ Ultrospec 2100 Pro UV/Vis spectrophotometer using a quartz cuvette with a 1-cm path length (with DPBS as blank). Calibration curves were constructed by plotting the absorbances both at 225 nm and 280 nm of each of the corresponding BSA concentrations (figure SI-7).

**Figure SI-7.** BSA calibration curve obtained upon albumin absorption at 225 nm and 280 nm (upper graphs) and the respective absorption peaks (lower graph).

Then, we proceed to quantify albumin and B_2_T release from the protein coated DMSNs. Given the absence of aromatic amino acids (*e.g.*, tryptophan (Trp) and tyrosine (Tyr)) in B_2_T peptide, the absorbance at 280 nm in BSA-DPBS medium is only due to albumin. Thus, the concentration of B_2_T in BSA-DPBS medium can be readily calculated from the absorbance value of B_2_T at 225 nm, which equals the total absorbance value at 225 nm minus the part of absorbance value belonging to BSA. Formulas used for this calculationare the followings:

***Abs^280nm^***_BSA_ *= X_1_C*_BSA_ + Z_1_,

***Abs^225nm^***_BSA_*= X_2_C*_BSA_+ Z_2_,

***Abs^225nm^***_B2T_ = ***Abs^225nm^*** *-* ***Abs^225nm^***_BSA,_

***Abs^225nm^***_B2T_*= X_3_C*_B2T_+ Z_3_.

where X_1_, Z_1_ are the given constants of the standard linear equation generated from BSA calibration curve at 280 nm; X_2_, Z_2_ are the given constants of the standard linear equation generated from BSA calibration curve at 225 nm; and X_3_, Z_3_ are the given constants of the standard linear equation generated from B_2_T calibration curve at 225 nm.

**Figure SI-8.** Calibration curve of B_2_T in BSA (250 µg/mL)-DPBS medium obtained with the UV-Vis spectrophotometer (A) and by HPLC (B). B_2_T concentrations in BSA-DPBS medium are 11, 22, 44, and 88 μg/mL.

*Method validation by HPLC*The reliability of our calibration curveswas confirmed using high-performance liquid chromatography (HPLC). We performed again the standard curve for B_2_T in BSA (250 μg/mL)-DPBS medium following our developed methodology with the UV-vis spectrometer and compared it to the standard curve obtained with HPLC. Results displayed in figure SI-8 confirm that our methodology is valid to detect B_2_T and albumin from mixed samples based on their distinct absorption at 225 nm and 280 nm, respectively.

*Albumin release from protein coated B_2_T@DMSNs-57 and B_2_T@DMSNs-156.*

Using the calibration curve obtained as explained in previous section, we managed to quantify the release of albumin from DMSNs surrounded by a protein corona concomitant to B_2_T release. 1.0 mg B_2_T@DMSNs (57 nm or 156 nm) were dispersed in 1.0 mL DPBS (pH 7.4) containing 250 μg/mL BSA. The samples were gently shaken at 37^o^C and, at predetermined time points, the suspension was centrifuged at 12,000 rpm for 10 min. We took the supernatant to measure the released albumin through its absorbance at 280 nm. The procedure was repeated for each time point and for both DMSNs. Fresh DPBS (same volume than aliquot of supernatant taken) was added to redisperse the pellet. All release measurements were performed in duplicate. Figures SI-9.A and SI-9.B show dynamic changes of BSA concentration in the medium during the B_2_T release test from B_2_T@DMSNs-57 and B_2_T@DMSNs-156, respectively. At 0.5 h, more than 150 μg/mL BSA was found in the supernatant. Within the first 138.5 h, all BSA is being released from the DMSNs. Afterwards, all BSA (250 μg/mL) was found in the supernatant.

**Figure SI-9.** Time dependent release of BSA from the surface of B_2_T@DMSNs-57 (A) or B_2_T@DMSNs-156 (B) to the medium.

# Section SI-2. Internalization of B_2_T@DMSNs by mice macrophages.

### Synthesis of fluoro-B_2_T@DMSNs.

200 µg BodiFluor-488 labelled B_2_T (designated as fluoro-B_2_T, Fig. SI-10.A) was dissolved in 0.4 mL DPBS buffer solution (pH 7.4), and the UV absorption spectra of this solution was measured by UV/vis spectrophotometer. Then, 1.0 mg DMSNs-56 or DMSNs-157 was added to the solution and the mixture was properly dispersed by sonication for 5 min. The resulting mixture was gently shaken at 200 rpm for 5 h at RT. Afterwards the products were separated by centrifugation at 12000 rpm for 10 min. The post-loading supernatant was also analyzed by UV/vis spectrophotometer.

Figure SI-10.B shows that after loading fluoro-B_2_T, white pellets of DMSNs became dark yellow. The characteristic peak (464nm) of fluoro-B_2_T was not visible in the supernatant after the loading process (Fig. SI-10.C) suggesting that fluoro-B_2_T was entirely loaded by both DMSNs-57 and DMSNs-156.

**Figure SI-10.** (A) General scheme of fluoro-B_2_T structure. (B) DMSNs pellets before and after fluoro-B_2_T loading. Colored pellets are visible after successful loading. (C) UV absorption spectra of fluoro-B_2_T before and after loading the DMSNs.

### Confocal laser scanning microscopy (CLSM) of cellular internalization.

Size and time dependent internalization of B_2_T@DMSNs was studied using RAW 264.7 cells from a macrophage-like Abelson leukemia virus transformed cell line derived from BALB/c mice. RAW 264.7 are regularly used as a macrophage cell model to study cell responses to microorganisms. We exposed the cells to fluoro-B_2_T@DMSNs (green) at different time points ranging from 0.5 to 16 hours. We labeled the cell membrane (magenta) to assess DMSNs internalization with help of CLSM. Figures SI-11 (B_2_T@DMSNs-57) and SI-12 (B_2_T@DMSNs-156) confirm time dependent DMSNs internalization.

**Figure SI-11. CLSM images of the internalization of fluoro-B_2_T@DMSNs-57 in Raw 264.7 macrophage cells.** Cells were exposed to the fluoro-B_2_T@DMSNs-57 from 0.5h to 16 h and imaged with a CLSM. The image shows the different channels obtained during imaging, bright field (cells in transmitted light), green channel (fluoro-B_2_T@DMSNs-57), magenta channel (cell mask dep red dye labeling the cell membrane) and an overlay of the green and the magenta channel. Scale bar 12 μm.

**Figure SI-12. CLSM images of the internalization of fluoro-B_2_T@DMSNs-156 in Raw 264.7 macrophage cells.** Cells were exposed to fluoro-B_2_T@DMSNs-156 from 0.5h to 16 h and imaged with a CLSM. The image shows the different channels obtained during imaging, bright field (cells in transmitted light), green channel (fluoro-B_2_T@DMSNs-156), magenta channel (cell mask dep red dye labeling the cell membrane) and an overlay of the green and the magenta channel. Scale bar 12 μm.

### Flow cytometry analysis of DMSNs internalization by Raw 264.7 macrophage cells.

Next, we show the FC data analysis of cellular-DMSNs interaction. RAW 264.7 cells were treated with fluoro-B_2_T@DMSNs-57 and fluoro-B_2_T@DMSNs-156 and with DAPI to distinguish dead from life cells. As presented in the histograms of figures SI-13 A and B, we first identified cells engulfing DMSNs based on their increased fluorescent signal compared to non-treated cells. We finally excluded dead (DAPI-stained) cells from the analysis, gated 5,000 individual cells engulfing DMSNs and obtained the mean fluorescent intensity (MFI) which we plotted in figure 3 of the manuscript.

**A**


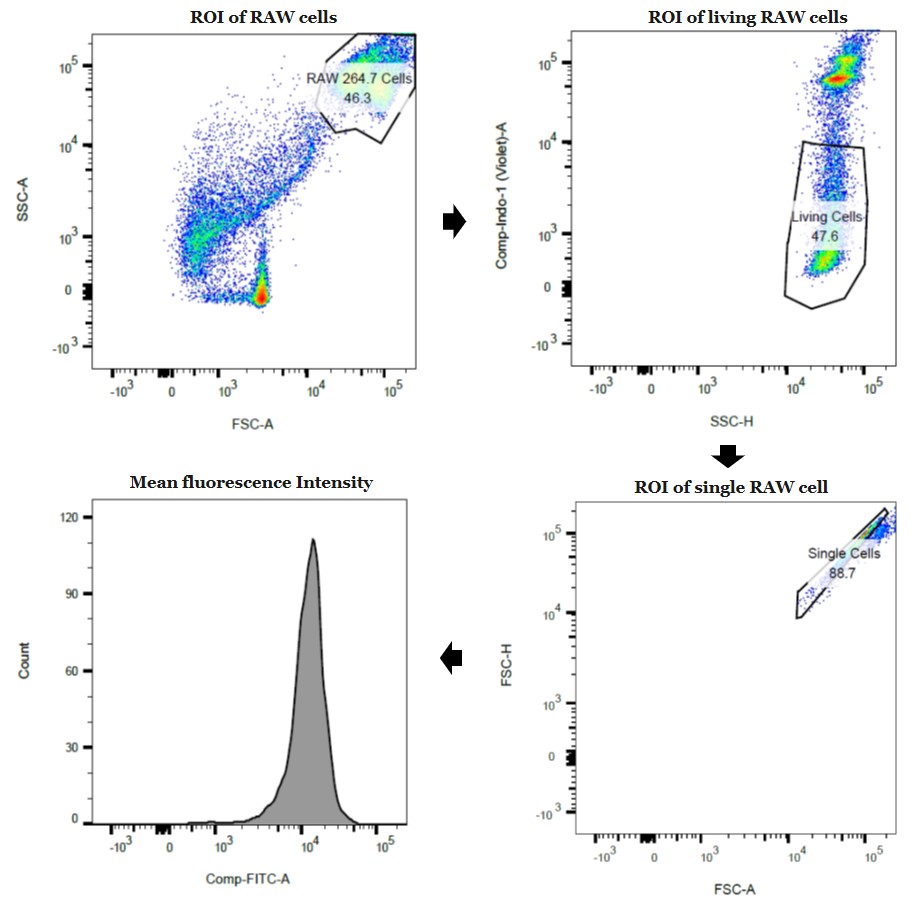


**B**

**
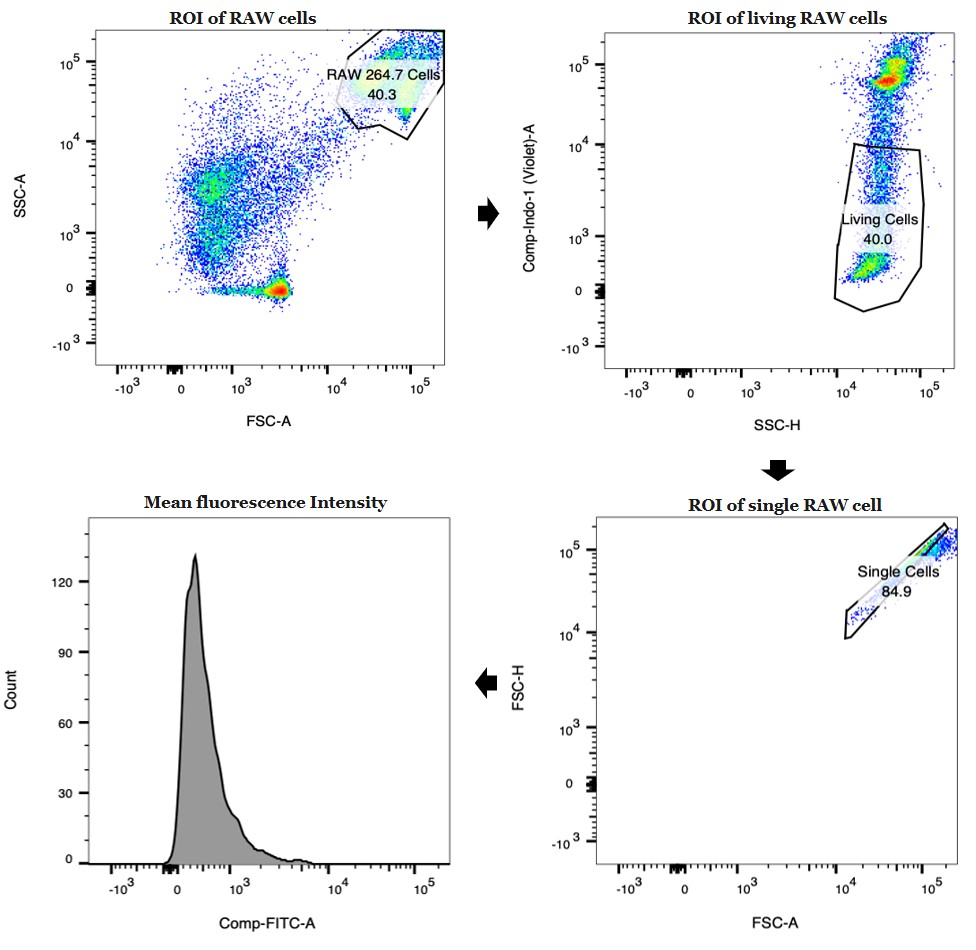
**

**Figure SI-13. Representative scheme of the flow cytometry internalization data analysis.** Raw 264.7 macrophage cells were exposed to fluoro-B_2_T@DMSNs-57 (A) or fluoro-B_2_T@DMSNs-156 (B) from 0.5 h to 16 h to quantify DMSNs internalization. Cells were labeled with DAPI to distinguish viable from non-viable cells. The figures represent a scheme of the data analysis. We followed the same procedure for all incubation time points.

# Section SI-3. Mice immunogenicity provided by B_2_T@DMSNs.

## Vaccination trials (I and II).

We performed two sets of *in vivo* experiments. In the first trial we wanted to confirm that the DMSNs were able to induce an immune response. Therefore, we treated mice with B_2_T@DMSNs and compared the response with bare DMSNs and with the positive control B_2_T@Montanide^TM^.

| **Treatment group** | **Number of mice tested** | **Sample** | **Injected dose** | **Equivalent B_2_T amount** |
| --- | --- | --- | --- | --- |
| **1** | **n = 4** | **B_2_T@Montanide^TM^** | **-** | **100 µg** |
| **2** | **n = 6** | **B_2_T@DMSNs-156** | **163 µg** | **100 µg** |
| **3** | **n = 4** | **DMSNs-156** | **163 µg** | **-** |


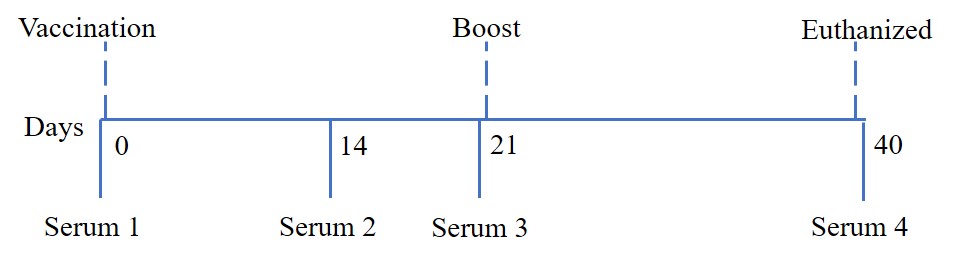


**Table SI-1. Vaccination trial I.** Scheme followed during the first *in vivo* vaccination trial. Vaccination of the different mice groups with the corresponding sample took place at day 0. After 21 days mice were boosted. Serum was collected at days 0, 14, 21, and 40. All mice were treated with the same amount of peptide vaccine.

In a second trial, we tried to elucidate if the immune response was nanoparticle size dependent. We also tried to evaluate if the antibody titers were sustained over time. Therefore, we treated mice with B_2_T@DMSNs-57 and B_2_T@DMSNs-156 and compared elicited antibodies up to 80 days post-vaccination. The positive control, B_2_T@Montanide^TM^, was also tested.

| **Treatment group** | **Number of mice tested** | **Sample** | **Injected dose** | **Equivalent B_2_T amount** |
| --- | --- | --- | --- | --- |
| **1** | **n = 3** | **B_2_T@Montanide^TM^** | **-** | **100 µg** |
| **2** | **n = 5** | **B_2_T@DMSNs-57** | **163µg** | **100 µg** |
| **3** | **n = 5** | **B_2_T@DMSNs-156** | **165µg** | **100 µg** |


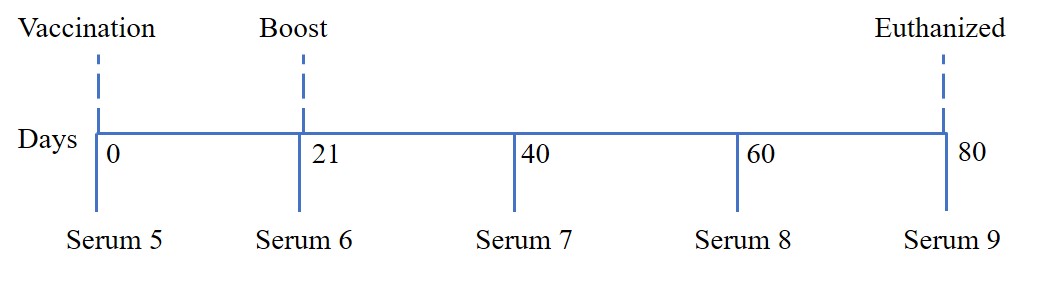


**Table SI-2. Vaccination trial II.** Scheme followed during the second *in vivo* vaccination trial. Vaccination of the different mice groups with the corresponding sample took place at day 0. After 21 days mice were boosted. Serum was collected at days 0, 21, 40, 60, and 80. All mice were treated with the same amount of peptide vaccine.

## Individual mice response to vaccination in trial II.

Results on figures (SI-14 - SI-16) reveal antibody responses (anti-B_2_T IgG titer) elicited by each mouse after the corresponding treatments: B_2_T@Montanide^TM^ (figure SI-14), B_2_T@DMSNs-57 (figure SI-15) and B_2_T@DMSNs-156 (figure SI-16). They show intra-animal variability of *in vivo* responses. For example, mouse 21 (M21) treated with B_2_T@Montanide^TM^ exhibited an IgG titer increase at day 21 (before the boost) (figure SI-14). This premature response was quite unusual since normally a second boost is needed to detect higher antibody titers (at 40 days).

**Figure SI-14. Individual mice response to B_2_T@Montanide^TM^.** M21, M22, M23 represent the response of three different mice.

In case of mice treated with B_2_T@DMSNs-156, 3 (M16, M17, and M19) out of 5 mice exhibited an immune response at day 21 (figure SI-16). Further studies are required to assess the significance of these results pointing out to an immunization before boosting. Note that M18 did not elicit a response until day 80. Mice treated with B_2_T@DMSNs-57 exhibited a more consistent response (figure SI-15).

**Figure SI-15. Individual mice response to B_2_T@DMSNs-57.** M24 to M28 represent the response of five different mice.

**Figure SI-16. Individual mice response to B_2_T@DMSNs-156.** M16 to M20 represent the response of five different mice.

# References.

[1] M.-J.C. van Lierop, J.M. van Noort, J.P.A. Wagenaar, V.P.M.G. Rutten, J. Langeveld, R.H. Meloen, E.J. Hensen, T cell-stimulatory Fragments of Foot-and-mouth Disease Virus Released by Mild Treatment With Cathepsin D, J. Gen. Virol. 75 (1994) 2937–2946. https://doi.org/10.1099/0022-1317-75-11-2937.

[2] E. Blanco, B. Guerra, B.G. De La Torre, S. Defaus, A. Dekker, D. Andreu, F. Sobrino, Full protection of swine against foot-and-mouth disease by a bivalent B-cell epitope dendrimer peptide, Antiviral Res. 129 (2016) 74–80. https://doi.org/10.1016/j.antiviral.2016.03.005.

[3] C. Cubillos, B.G. de la Torre, A. Jakab, G. Clementi, E. Borrás, J. Bárcena, D. Andreu, F. Sobrino, E. Blanco, Enhanced Mucosal Immunoglobulin A Response and Solid Protection against Foot-and-Mouth Disease Virus Challenge Induced by a Novel Dendrimeric Peptide, J. Virol. 82 (2008) 7223–7230. https://doi.org/10.1128/jvi.00401-08.

[4] J. Tu, A.L. Boyle, H. Friedrich, P.H.H. Bomans, J. Bussmann, N.A.J.M. Sommerdijk, W. Jiskoot, A. Kros, Mesoporous Silica Nanoparticles with Large Pores for the Encapsulation and Release of Proteins, ACS Appl. Mater. Interfaces. 8 (2016) 32211–32219. https://doi.org/10.1021/acsami.6b11324.
